# Luminal endothelialization of small caliber silk tubular graft for vascular constructs engineering

**DOI:** 10.3389/fcvm.2022.1013183

**Published:** 2022-11-16

**Authors:** Stefano Rizzi, Sara Mantero, Federica Boschetti, Maurizio Pesce

**Affiliations:** ^1^Centro Cardiologico Monzino, IRCCS, Milan, Italy; ^2^Ph.D. Program in Biomedical Engineering, Politecnico di Milano, Milan, Italy; ^3^Department of Chemistry, Materials and Chemical Engineering “Giulio Natta”, Politecnico di Milano, Milan, Italy

**Keywords:** tissue engineered vascular graft, cardiovascular tissue engineering, high seeding efficiency, silk fibroin scaffold, *in vitro* dynamic cell seeding and culture, bioreactor for vascular tissue engineering endothelialization of silk tubular scaffolds

## Abstract

The constantly increasing incidence of coronary artery disease worldwide makes necessary to set advanced therapies and tools such as tissue engineered vessel grafts (TEVGs) to surpass the autologous grafts [(i.e., mammary and internal thoracic arteries, saphenous vein (SV)] currently employed in coronary artery and vascular surgery. To this aim, *in vitro* cellularization of artificial tubular scaffolds still holds a good potential to overcome the unresolved problem of vessel conduits availability and the issues resulting from thrombosis, intima hyperplasia and matrix remodeling, occurring in autologous grafts especially with small caliber (<6 mm). The employment of silk-based tubular scaffolds has been proposed as a promising approach to engineer small caliber cellularized vascular constructs. The advantage of the silk material is the excellent manufacturability and the easiness of fiber deposition, mechanical properties, low immunogenicity and the extremely high *in vivo* biocompatibility. In the present work, we propose a method to optimize coverage of the luminal surface of silk electrospun tubular scaffold with endothelial cells. Our strategy is based on seeding endothelial cells (ECs) on the luminal surface of the scaffolds using a low-speed rolling. We show that this procedure allows the formation of a nearly complete EC monolayer suitable for flow-dependent studies and vascular maturation, as a step toward derivation of complete vascular constructs for transplantation and disease modeling.

## Introduction

Ischemic heart disease remains, both in the European Union and in the US the most represented cause of death in elderly people according to the latest reports. The pathology has a continuously increasing trend in the rest of the world (1, 2). Despite the use of modern interventional cardiology procedures and the adoption of drug-eluted stents has made reperfusion of the ischemic heart an extremely safe and effective practice, recurrence of ischemic events by “in-stent” restenosis (3) makes necessary the adoption of surgical revascularization, which consists of coronary artery bypass grafting (CABG) (4). CABG procedures employs autologous arteries (e.g., the radial or the mammary arteries) or the great saphenous vein (SV) to restore perfusion to tissues downstream the occlusion, especially in multi-vessel coronary artery disease (5, 6). Compared to artery-made bypasses, arterialized SV conduits exhibit shorter term patency (around 50% of failure at 10 years after implantation) mainly due to a maladaptive remodeling processes involving proliferation of smooth muscle cells (SMCs) in the intima layer, and causing reduction of vessel patency (4, 7). Various causes of SV grafts failure have been discussed in the literature depending on the timing of bypass occlusion. At early stages, SV conduits fail for thrombosis events, while at later times due to intimal hyperplasia and graft atherosclerosis (4). Different causes account for these failure modalities. The precocious thrombotic events are principally due to vein denudation, which occur in consequence of vessel harvesting, pre-implantation storage and implantation modalities (6, 8). Neo-intima accumulation, instead, consist of a phase of progressive growth of SMCs in the intima layer that is prompted by accumulation of inflammatory cells (9) and mechanical cues (10); this growth reduces the patency of the grafts over time and makes them liable to lipid accumulation and secondary atherosclerosis (6).

With the perspective of a growing clinical demand in this area, there is an intense ongoing research to manufacture definitive tissue engineered vessel grafts (TEVGs) endowed with the ability to grow and self-renew. In fact, despite several fully engineered vessels have been described in the literature (11, 12), the problem of the post-implant remodeling remains actual (13). In this respect, one of the factors that seems to be crucial to reduce the remodeling of the natural bypass conduits and, potentially, TEVGs, is the presence of a fully differentiated endothelial layer, functioning as a barrier to the homing of inflammatory cells and producing NO, a molecule with potent anti-inflammatory (14, 15) and vaso-relaxing activity (14). The presence of a functional endothelial layer is finally also necessary to optimize hemocompatibility and reduce the risk of thrombus formation in the conduits that may lead to sudden closure (6, 8).

In the present contribution, we employed a new strategy to create a uniform endothelial layer onto the luminal surface of electrospun silk tubular scaffolds using a low speed rolling bioreactor. By showing the feasibility of silk endothelialization with this method, and the resistance of the endothelial layer to the application of a steady flow due to the formation of a basal membrane, our work paves the way toward a standardized method for manufacturing TEVG for flow-dependent studies and vein/arterial *in vivo* replacement in CABG procedures.

## Methods

### Vascular scaffold manufacturing

Silk fibroin was chosen as a material for scaffold manufacturing because of its good mechanical and biocompatibility properties. Commercial tubular scaffolds were produced by electrospinning (Leonardino s.r.l., Italy) consisting at first of dissolution of silk fibroin films in formic acid 98–100% (Sigma Aldrich) for 20 min at room temperature (16). The nanofibrous scaffold was then obtained by electrospinning of the silk fibroin solution on the cylindrical mandrel, using needle EF300 (SKE Research Equipment). After letting the solvent completely evaporate, cylindrical scaffolds were briefly treated with methanol and sterilized in ethylene oxide, obtaining a device with an average wall thickness of 100–150 μm and a fiber diameter in a range of 400–800 nm. All the scaffolds were delivered in a tubular geometry of 0.5 cm in diameter, for their similarity to a human coronary blood vessel, and 10 cm in length.

### Scaffolds characteristics and preparation/coating

To characterize the silk fibroin scaffolds, permeability tests on the scaffold were performed with an apparatus made of two coaxial stainless-steel cylindrical parts and a capillary flow meter with a resolution of 1 μL. Round-shaped silk specimens, of 10 mm in diameter, were housed into the lower cylinder, over a polyethylene porous filter, and secured between two gaskets. The permeability factor K was then calculated as defined by Darcy’s law:


$$\text{K}=\frac{QL}{A\Delta p}$$

where fluid with a pressure (Δp) ranging between 1470 Pa and 7350 Pa was imposed. After tissue equilibration, time necessary to filter 10 mm^3^ of water was measured.

Scaffold porosity was evaluated according to the following formula (17):


$$\%P=100\left(1-\frac{M}{V\rho }\right)$$

considering a silk fibroin scaffold sample of mass (M) equals to 0.0985 g, geometry of: 1.8 cm in length, inner diameter of 0.5 cm and outer diameter of 0.625 cm and density of 1.35 g/cm^3^ (17).

For static/dynamic seeding and culture, the matrices were downsized to the desired size and shape and rinsed extensively in PBS. Subsequently, scaffold samples for the static and the dynamic cell seeding and culture were incubated in FBS overnight at 37°C in order to promote adsorption of adhesive proteins over the seeding surface.

### Cells

Endothelial cells (EA.hy926; ATCC) cells were expanded for several passages in dishes previously coated with gelatin 0.2% and basic culture medium (DMEM, 10% fetal bovine serum, 1% penicillin) further supplemented with 1% Non-Essential Amino Acids Solution (Thermo Fisher, USA), 1% Tricine Buffer 1 M pH7 (Sigma-Aldrich) and HAT Media Supplement (50 ×) Hybri-Max (Sigma-Aldrich).

### Bioreactor utilized

**FIGURE 1:**
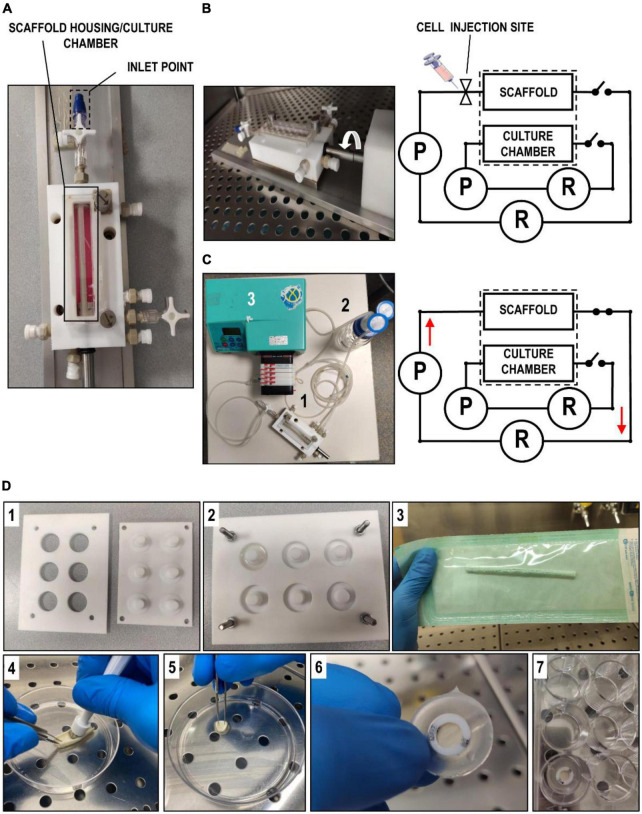
Illustration of bioreactors and scaffolds derivation. **(A)** Overall hydraulic scheme of the Minibreath bioreactor. It is shown the compartment used for scaffold housing and the inlet point where cells were injected to proceed with seeding. **(B)** The picture on the left and the scheme on the right show, respectively, an illustration of the rotating seeding phase and the configuration of the bioreactor. P indicates the peristaltic pump and R the medium reservoir. During the seeding phase, cell suspension was injected inside the luminal compartment of the scaffold after which, the system was allowed to rotate the scaffold for a period of 3 days. **(C)** After 3 days of seeding phase, the scaffold was perfused with a physiological venous flow (0.5 mL/min) provided by a closed loop hydraulic circuit (scheme on the right), consisting of tubing (1), a reservoir (2) and a peristaltic pump (3). **(D)** Description of the tools and the procedure employed to optimize the seeding of the silk scaffolds. Panels 1 and 2 show, respectively, the molds used to fabricate the PDMS holders used to house the circular silk patches in cell culture, while panel 3 shows one of the tubular scaffolds still in its sterile packaging. Panels 4–7 contain a sequence of pictures illustrating how we derived circular patchs from a tubular scaffold and placed them in a multiwell culture plate after housing into the PDMS mold. Static cell seeding and culture experiments were performed by gently pipetting ECs suspension over pre-wetted scaffold.

The system used for dynamic seeding and culture was composed of the Minibreath bioreactor housed inside its drive motor base plate and comprehensive of Control Unit and Motor Drive (Harvard Apparatus Regenerative Technology) and a peristaltic pump (Watson-Marlow SciQ 323, head type: 314 MC). This device allows a physical separation between the inner volume of a tubular scaffold and the outside environment. Furthermore, the possibility to house in the bioreactor’s chamber the tubular scaffold and to connect it with a hydraulic circuit, enables cell seeding by slow-rotation of the scaffold luminal surface and the creation of a recirculation flow of a fluid from a reservoir by means of a chosen type of pump, in our case: peristaltic (Figure 1).

### Static cells seeding

For testing the ideal ECs concentration to cover the surface of the scaffolds, we set seeding experiments onto flat round silk scaffolds (1 cm in diameter) produced by cutting the tubular scaffolds with a puncher under sterile conditions (Figure 1). Subsequently, scaffolds mounted onto PDMS holders with rigid o-rings placed inside a 12 culture well plate (inner surface of the tubular scaffold upturned). PDMS holders were specifically prepared in suitable molders (Figure 1). A total of 300 μl volume culture medium was applied onto the free surface of the scaffolds to allow cell adhesion, after soaking the scaffolds with the same culture medium used for the culture. Two hours after seeding, an additional mL of fresh medium was added on top of the seeded scaffold. The optimal cell concentration was established during this phase by seeding different cellular quantities; 75 × 10^3^, 150 × 10^3^, 300 × 10^3^, 600 × 10^3^, 900 × 10^3^ cells per scaffold. For each cellular density, experiments were repeated at least three times.

### Dynamic cell seeding/culture

After tubular scaffold preparation in FBS as described above, samples were downsized to 3.5 cm in length and then rinsed abundantly with PBS, before placing in.in the Minibreath bioreactor’s chamber. A total of 2.1 × 10^6^ endothelial cell (EC)/scaffold were then injected in the inner chamber of the scaffold (Figure 1) and left for 3 days under continuous rotation at 1.5 RPM. To test the adhesion of the seeded cells to the internal surface, a sample of approximately 1.5 cm in length was collected from the seeded scaffolds using MTT assay. Subsequently, a hydrodynamic circuit, composed of a peristaltic pump (Watson-Marlow SciQ 323, pump’s head: 314 MC), tubings (PharMed), connectors (BD Connecta) and a 200 mL reservoir were connected to the inner luminal compartment to dynamically stimulate the cells with a flow of cell culture medium at a rate of approximately 0.5 mL/min for additional 3 days (Figure 1).

### Cells viability assays, histological sectioning and staining, immunohistochemistry, and microscopy analyses

At the end of the experiments, the scaffolds were harvested, cut along their perpendicularly axes and used, alternatively, again for MTT assay or fixation in 4% paraformaldehyde, for microscopic analyses (half of the sample). Samples employed for histological analyses and immunohistochemistry were embedded in OCT (Bio-Optica, Italy) for frozen-sectioning and staining with hematoxylin and eosin or staining with anti-Laminin antibody (Abcam, UK) at a concentration of 7 μg/mL, followed by incubation with a secondary HRP-conjugated antibody (concentration: 10 μg/mL in blocking buffer consisting of phosphate-buffered saline with, 3% bovine serum albumin, and 0.1% Triton X-100) and the final staining with DAB. Images were acquired with an Axioscop optical microscope (Carl Zeiss). Cellularized scaffolds used for scanning electron microscopy analyses were dehydrated with a 50, 75, 95, and 100% ethanol series and subsequently sputter-coated with gold. From each sample we acquired fifteen images with a Stereoscan 360 scanning electron microscopy (Cambridge Instruments, Cambridge, UK), by ideally subdividing the overall area of the flattened scaffold in left, central and right area and considering five images of the three areas.

### Statistical analysis

All the images obtained by scanning electron microscopy were analyzed and the percentage of covered and uncovered area of the inner scaffold surface was calculated with ImageJ software (National Institute of Health). In addition, images of the unseeded scaffold were evaluated in superficial porosity by considering and computing the area of the superficial pores present in the inner part of the scaffold. All values in bar graphs are represented as mean ± standard error (SE). Differences among experimental groups were assessed by GraphPad. Type of statistical tests and number of replicates included in the analyses are specified in the figure legends.

## Results

### Scaffold characterization, functionalization, and ECs basal adherence

Scaffold permeability evaluation resulted in an average value of 1.64e^–12^ ± 6.98e^–14^ m^2^; mean ± SE, *n* = 5. This value was considered to be comparable to permeability values of natural polymers (18). The evaluation of scaffold porosity indicated an overall value of 90.82%, in line with porosity values of silk fibroin scaffold (19–23). Imaging by SEM, finally, revealed an average surface porosity of 27.4% ± 3.91% (mean ± SE, *n* = 4) and an average pore size of 5.85 ± 1.45 μm^2^, mean ± SE, *n* = 5).

**FIGURE 2:**
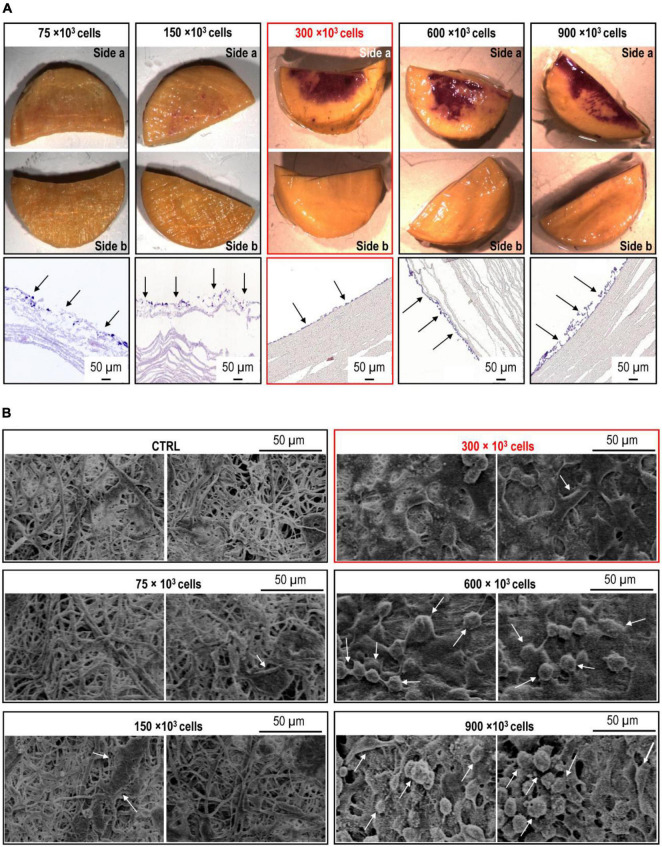
Optimization of ECs seeding onto circular silk scaffold patches. **(A)** Increasing amounts (75 × 10^3^–900 × 10^3^) of cells were seeded onto the circular silk scaffold patches produced as in Figure 1D. The pictures on the upper side of the panel show the MTT staining of both sides of the scaffold halves, where the increasing purple color indicates an increased coverage of the seeding surface (side a). The lack of staining on sides b indicates that there was no migration of the cells on the opposite scaffold side. The micrographs on the bottom part of the panel show the appearance of the endothelial cell (EC) layer as observed in transversal section of the scaffold. It is evident the formation of an EC monolayer at 300 × 10^3^ ECs concentration. **(B)** SEM micrographs of the sides a of the silk scaffold circular patches cellularized with the increasing amounts of ECs. Also in this case, 300 × 10^3^ was the optimal cell concentration, favoring the formation of a monolayer made of firmLy adhering cells.

Static cell seeding experiments were initially performed using silk circular scaffolds in the absence of coating to assess the basal ability of endothelial cells to adhere to the electrospun silk material. These experiments did not produce good results (data not shown) due to the poor adherence of the cells. To overcome this problem, we adopted a simple coating process of the planar/tubular scaffolds by immersing them into FBS overnight at 37°C. After preliminary tests showing a higher adhesion of the cells to the FBS-treated scaffolds, we optimized the cell quantity to allow formation of a uniform endothelial layer over the functionalized scaffolds. The MTT cell viability assay was chosen to obtain a colorimetric indication of the cell distribution over the scaffolds, as already described by us for another scaffold cell seeding application (24). Transversal sectioning and scanning electron microscope observation of the cellularized planar scaffolds were also used to verify the formation of an endothelial monolayer. As shown in Figure 2, from these experiments the 300 × 10^3^ cells/cm^2^ concentration emerged as the minimal amount of cells to ensure a nearly complete coverage of the scaffold lumen with ECs. Stronger indications that this concentration was the best came from the results of histological transversal sectioning and of SEM observations. Indeed, at higher concentrations (e.g., 600.10^3^ and 900.10^3^ cells/cm^2^) ECs tended to pile up and formed a multilayered endothelial sheet with no benefit for the increase of lumen coverage (Figure 2). In light of these results, we assumed 300.10^3^ cells/cm^2^ as be cell density to be used in subsequent experiments.

### Flow stimulation of the endothelial layer induces morphological changes in ECs and promotes formation of a basal lamina

**FIGURE 3:**
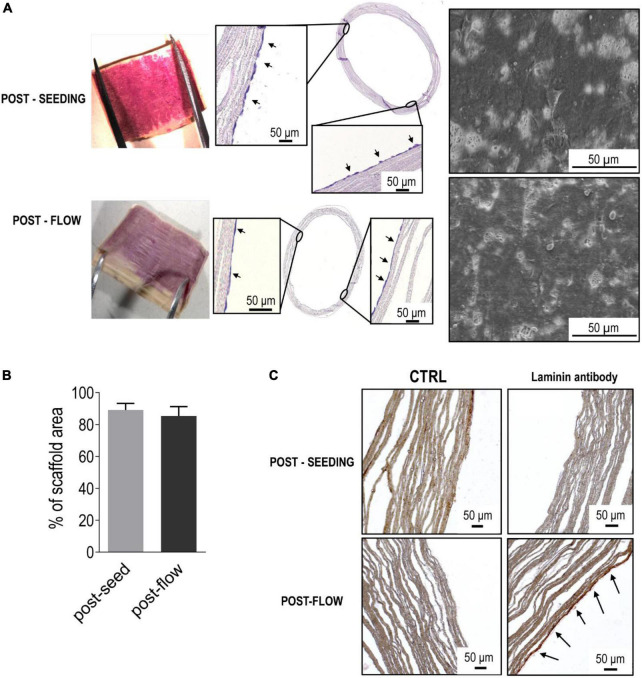
Efficiency of the rotation-dependent ECs seeding and firm adhesion of ECs under a slow flow. **(A)** The picture on the top-left of the panel shows a nearly complete coverage by ECs of the luminal scaffold surface, as detected by MTT assay. The micrographs on the center and the right indicate, respectively, the structure of the EC layer in transversal section and the spreading of the cells onto the silk scaffold. Application of a constant flow (0.5 mL/min for 3 days), did not produce substantial modifications in the ECs coverage, neither at a macroscopic level (MTT staining and transversal sectioning), nor at a microscopic observation. This witnesses a good compatibility of the scaffold for firm adhesion of ECs resistant to application of a steady flow. Quantification of the area covered by ECs onto SEM micrograph showed that the cell coverage did not decrease in presence of a flow **(B)**, and that cells exposed to flow clearly synthesized a basal lamina, to reinforce their own firm adhesion **(C)**.

To assess the robustness of EC attachment on the luminal surface of the scaffold, we applied a laminar flow of 0.5 mL/min for 72 h. Considering that the shear stress τ at the wall of a tube of diameter *D* can be calculated as:


$$\tau =\frac{8\mu v}{D}$$

where μ is the viscosity of the culture media (0.01 g/cm. s^–1^) (25) and *v* is the velocity of the fluid inside the tubular conduit (26), the resultant shear stress perceived by the cells was about 0.007 Pa (that equals to 0.07 dyne/cm^2^). Determination of the lumen coverage by ECs was again performed by MTT assay and SEM. This was done in portions of the scaffolds after the seeding period, and for comparison in adjacent portions of the same scaffolds subjected to flow for 3 days (see section “Methods”). As shown in Figure 2A, MTT assay did not exhibit a reduction in the staining on the lumen of the scaffolds subjected to flow, suggesting that the cells had resisted to the flow shear stress. On the other hand, the appearance of the cells was different at pre- and post-flow stimulation stages, with a flatter morphology in flow-treated scaffolds vs. the corresponding controls (Figure 2B). SEM observations confirmed the flatter morphology of the adherent ECs and a similar coverage of the scaffold lumen before and after application of the flow (Figure 3A). Evidences in the literature suggest that application of flow shear forces to endothelium induces formation of a basal lamina (27, 28). In order to understand whether this happened also in our samples, we performed immunohistochemistry on transversal sections of the endothelialized scaffolds, before and after exposure to flow. This clearly showed that flow induced cells to deposit Laminin, suggesting a mechanical maturation of the endothelial layer (Figure 3).

## Discussion

Synthetic and natural biomaterials have been both assessed for engineering blood vessels. While synthetic materials (such as PGA and PCL) offer, at least in principles, tunability of mechanical properties and controlled degradation over time, their relevance *in vivo* is still limited, especially when considering graft patency duration (29). Decellularized matrices (30, 31) have been also considered to this aim, but these materials still suffer from logistical hurdles, given the limited donor-to-donor availability. Furthermore they do not maintain patency at long term due to a non-complete detoxification of the agents used to decellularize them (32, 33), the failure at completely removing major xenoantigens, or mechanical discontinuities at the anastomoses between the native blood vessel and the intercalated implant that favor neointima accumulation (34). By contrast, natural polymers offer the possibility to overcome some of these drawbacks, but often lack adequate mechanical characteristics (35).

Silk fibroin holds great promise for the fabrication of functional TEVGs considering its excellent biocompatibility, a natural anti-thrombogenicity, the low generation of inflammatory responses, the possibility of blending with other biocompatible materials (e.g., PCL and PLA) (36–40, 41). Moreover, numerous functional modifications of silk fibroin are possible, to add peculiar manufacturing characteristics, such as, for example, to finely tune the protein surface chemistry and hydrophilicity for an optimal cell attachment and proliferation (20). Finally, thanks to availability of various procedures to depose silk fibroin fibers (e.g., 3D printing or electrospinning), it is relatively easy to obtain three-dimensional structures such as tubular scaffolds able to reproduce the geometrical characteristics of blood vessels (41, 42). The chosen electrospinning process, in particular, exploited the use of formic acid as solvent during scaffold fabrication to ensure maximal biocompatibility and retention of bioactivity and morphology of the scaffold’s structure (43). The compliance of these tubular scaffolds have also a mechanical advantage compared to equivalent tubular structures with similar dimension, with a 2.4%/100 mmHg of diameter compliance for silk electrospun graft vs. 2%/100 mmHg of diameter compliance for P(LLA-CL) tubular structures with similar geometry (40).

In the search for innovative solutions to replace the currently employed autologous vessels in vascular/cardiac surgery, various approaches have been attempted. These range from simple cell-seeding approaches followed by graft maturation in culture (44) to wrapping cells sheets around a mandrel in order to fabricate engineered tubular structures (45). Despite enormous advances and various attempts, to date no definitive solution exists able to go beyond the phase of the early clinical trials (46). As a first step toward the derivation of a procedure to produce fully tissue-engineered small caliber vascular grafts able to surpass the currently adopted solutions, we set up a controlled endothelialization method of electrospun silk tubular scaffolds *via* a two-step procedure consisting of: (i) a cell seeding procedure by rolling of the tubular scaffold previously soaked overnight in FBS at 37°C; and (ii) application of a low flow rate to the cell seeded tubular scaffold to improve EC attachment to the scaffold. In this respect, it is noticeable that in analogy with existing approaches, where the porosity of silk tubular scaffolds occupies a good fraction of the scaffold volume (∼80%), our scaffold had a porosity of ∼90% thus, at least in principles, facilitating cell invasion (19–23). Interestingly, the superficial pore density of our scaffolds amounted to <30% of the total surface with an average pore dimension of ∼6 μm^2^. Although this relatively low pore size might would not compromise cell invasion from the surface of the scaffold, it probably offered the cells an optimal density of attachment sites for the formation of a monolayered EC sheet, as it was also suggested by the deposition of a basal lamina (Figure 3). On the other hand, at least *in vivo*, cellular colonization of scaffolds with a fiber density similar to that employed in our study [see for example ref (47)], occurs not only due to direct penetration of the cells in the interleaved spaces among fibers, but also due to the ability of the cells to reabsorb the material and substitute it with cell-derived extracellular matrix components.

Despite this work is not the first to propose a low speed rolling procedure for endothelial cells seeding and adherence onto the lumen of a bioartificial scaffold–see for example (21), it is the first to demonstrate the necessity to tightly control the efficiency of cell seeding using quantitative criteria. Our results, in fact, show that the overall confluence of the endothelial layer can have a relevant impact on the adhesion/spreading efficiency of the cells, making the endothelial layer subjected to potential variations in shear stress in correspondence of non-completely confluent or multilayered endothelium, thus giving rise to partial or total detachment of the endothelial layer over time or, more in general, to an inhibition of the endothelium function (48, 49). These findings are confirmed by the experiments performed in the presence of a steady flow, in which the mono-layer of ECs adhering to the scaffolds resisted to the application of 0.5 mL/min of flow and were induced to secrete a basal lamina (27). Of course, experiments will be necessary in the future to assess whether the ECs are induced to further maturation by application of consecutive increments of shear stress up to the physiological level present, for example in the normal veins due a steady flow that is 0.1 Pa or 1 dyne/cm^2^ (50), or to an oscillating shear stress such as that experienced by the ECs in coronary arteries that is 1–7 Pa, i.e., 10–70 dyne/cm^2^ (51–53).

In summary, with this work we overcome some of the issues related to current TEBV fabrication methodology, and in particular the lack sufficient biomimicry of the endothelial cell layer, which jeopardizes the full biocompatibility and, prospectively, the long-term patency of the grafts (46). This strategy appears optimal to seed autologous or immunologically compatible endothelial cells for “personalized” or allogenic grafting after evaluation of donor/recipient immunological compatibility. Further studies are finally necessary to assess whether the EC layer realized on the scaffold lumen withstands the normal flow/pressure regimens of the venous and coronary circulation; the final biocompatibility of the grafts using *in vivo* models; and the economic sustainability of grafts translation in vascular/cardiac surgery.

## Data availability statement

The raw data supporting the conclusions of this article will be made available by the authors, without undue reservation.

## Author contributions

SR, SM, and MP conceived the main conceptual idea and planned the experiments. SR performed the experiments and wrote the original draft. SR analyzed the data under the supervision of MP and SM with the contribution of FB. SM revised the first draft and contributed to the organization of the figures. MP revised the final draft. All authors have read and approved the version of the manuscript to be published.
